# Candida Epiglottitis in a Patient Undergoing Chemotherapy for Small Cell Lung Cancer: A Case Report

**DOI:** 10.7759/cureus.72607

**Published:** 2024-10-29

**Authors:** Natsumi Kushima, Toyoshi Yanagihara, Takato Ikeda, Maiya Chen, Naoki Hamada, Masaki Fujita

**Affiliations:** 1 Department of Respiratory Medicine, Fukuoka University Hospital, Fukuoka, JPN

**Keywords:** acute epiglotitis, cancer management, candida epiglottitis, febrile neutropenia, immunocompromised

## Abstract

Epiglottitis is a critical infection that can result in upper airway obstruction. While bacterial infections are the most common cause of acute epiglottitis, Candida epiglottitis remains relatively rare. We report a case involving an 82-year-old male undergoing chemotherapy for small cell lung cancer. The patient had a history of chronic obstructive pulmonary disease, which was managed with long-term inhaled corticosteroids. On the 10th day after receiving amrubicin and pegfilgrastim, he developed fever and a sore throat, presenting to the clinic the following day. Clinical examination revealed white patches on the palate, a muffled voice, and inspiratory wheezing. Laboratory tests indicated severe neutropenia. Laryngoscopy confirmed epiglottic swelling with white pseudomembranes, leading to a diagnosis of Candida epiglottitis. The patient was hospitalized and treated with intravenous micafungin and meropenem, along with amphotericin B gargles. Symptoms improved rapidly, with the resolution of fever and sore throat by the next day. Antifungal therapy was completed within 11 days of hospitalization. This case underscores the importance of considering Candida epiglottitis in immunocompromised patients presenting with a sore throat, as prompt diagnosis and treatment are essential to prevent potentially fatal complications.

## Introduction

Epiglottitis is an acute inflammation of the upper larynx that involves the epiglottis. It typically presents with a sore throat and can progress to dysphagia, hoarseness, and potentially life-threatening dyspnea. When respiratory distress occurs, it becomes a medical emergency requiring immediate and precise airway management. While various pathogens can cause epiglottitis, most are endemic to the oral cavity, including Candida species. Although Candida epiglottitis is rare, it has been documented in immunocompromised individuals, particularly those undergoing cytotoxic chemotherapy, suffering from viral infections (e.g., cytomegalovirus), experiencing neutropenia, or diagnosed with acquired immunodeficiency syndrome [1,2]. Prolonged use of broad-spectrum antibiotics and steroids also increases the risk, with inhaled corticosteroids being a significant contributing factor to this infection [3].

Diagnosis relies on a combination of clinical presentation, local findings, histopathological examination, and bacteriological testing [4]. Characteristic local findings include white, plaque-like lesions on the laryngeal mucosa. While histopathological examination is crucial for differentiating Candida epiglottitis from conditions such as leukoplakia, malignancy, tuberculosis, or syphilis [4], the rapid response to antifungal treatment often allows for diagnosis based solely on clinical course and endoscopic findings [3].

Candida epiglottitis requires particular vigilance in immunocompromised patients, especially those with neutropenia, due to the risk of progression to severe respiratory infections or systemic candidiasis [1]. Prompt intervention with antifungal treatment and respiratory support is often necessary. This report presents a case of Candida epiglottitis that developed concurrently with chemotherapy-induced neutropenia in a patient with lung cancer, highlighting the importance of considering fungal etiologies in at-risk populations presenting with upper airway symptoms.

## Case presentation

An 81-year-old man with recurrent small-cell lung carcinoma presented with a one-day history of odynophagia and fever (38 °C) on day 11 of third-line amrubicin chemotherapy and pegfilgrastim administration. He was unable to eat anything due to a severe sore throat. His medical history included chronic obstructive pulmonary disease (COPD), non-valvular atrial fibrillation, and previous surgeries for rectal and prostate cancers. He had a 40-year history of smoking seven cigarettes per day and used inhaled fluticasone furoate, umeclidinium bromide, and vilanterol trifenatate (dry powder) for COPD management.

**Figure 1:**
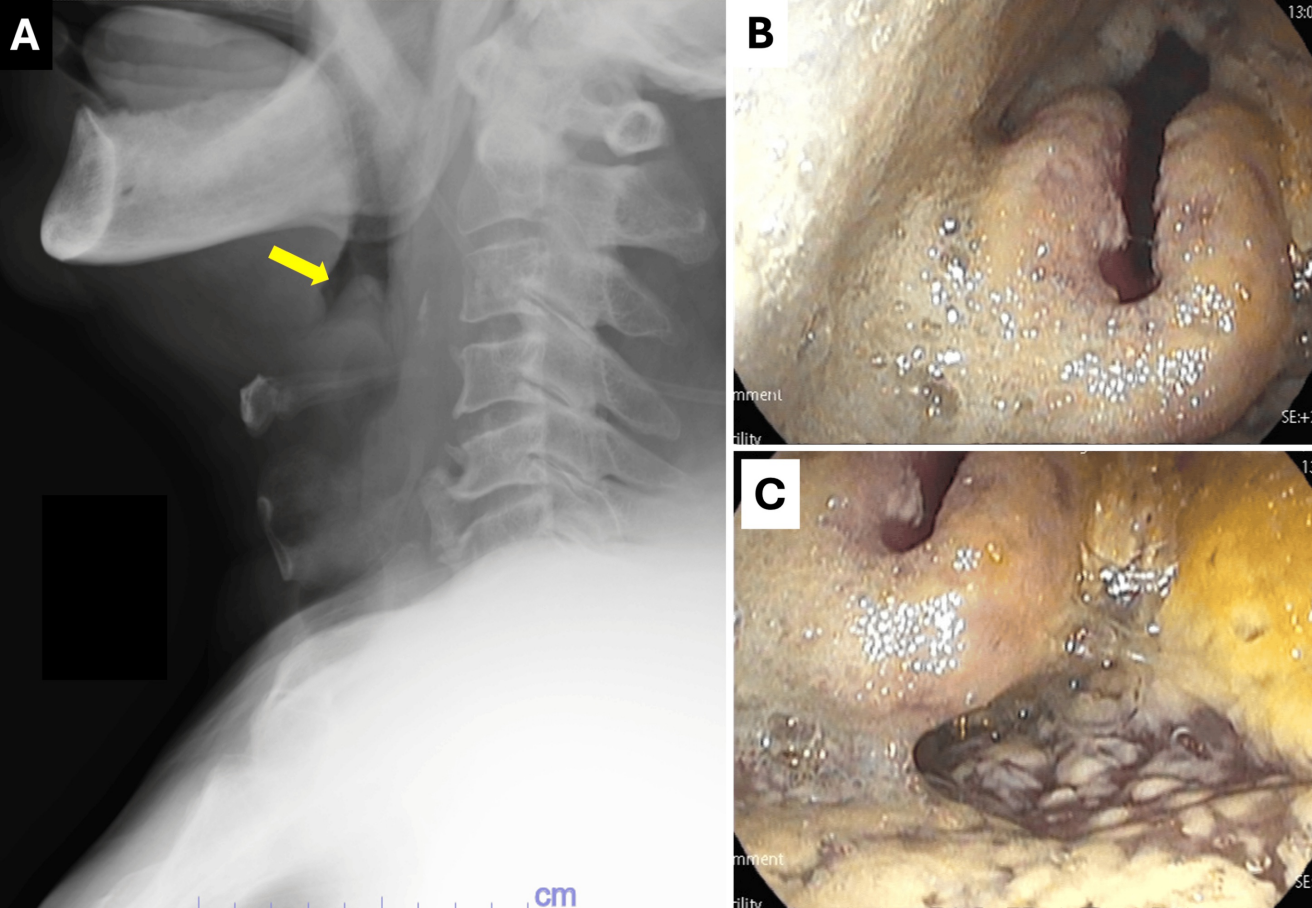
Diagnostic imaging and endoscopic findings of Candida epiglottitis (A) Lateral neck X-ray demonstrating swelling of the epiglottis, exhibiting the classic “thumb sign” characteristic of epiglottitis (indicated by an arrow). (B, C) Laryngoscopic views of the oropharynx revealing extensive white pseudomembranous lesions, along with swelling of the epiglottis and surrounding tissues, consistent with Candida epiglottitis.

On examination, his vital signs were as follows: pulse rate 109 beats per minute, blood pressure 136/77 mmHg, temperature 37 °C (after antipyretics), respiratory rate 22 breaths per minute, and SpO2 91% on room air. He was alert and oriented. No abnormalities were noted in the tongue or buccal mucosa, but several white patches were observed on the palate. Auscultation revealed inspiratory wheezes and a muffled voice. Laboratory tests showed a white blood cell count of 300/μL, a neutrophil count of 25.5/μL (decreased), and an elevated CRP of 24.38 mg/dL. Both β-D-glucan (<6 pg/dL) and Candida mannan antigen (<0.02 U/mL) were within normal ranges. Chest X-ray showed no new abnormalities aside from the known lung cancer mass, while lateral neck X-ray revealed swelling of the epiglottis (thumb sign) (Figure 1A).

Laryngeal fiberoscopy, performed due to suspected acute epiglottitis, revealed white pseudomembranous patches covering the epiglottis and extending from the pharynx to the upper part of the epiglottis. The epiglottis exhibited moderate swelling, which extended to the laryngeal surface and presented a U-shape (Figure 1B, 1C). A throat swab for fungal culture was not obtained, as candidiasis was diagnosed based on the evident signs of white patches. Given the concern for upper airway obstruction during the acute phase, it was determined that taking additional risks was unnecessary. Based on these findings, a diagnosis of Candida pharyngolaryngeal candidiasis with epiglottitis and febrile neutropenia was established, necessitating emergency hospitalization.

**Figure 2:**
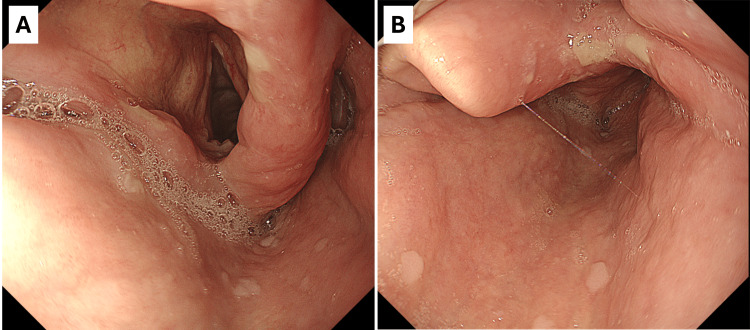
Endoscopic views of the larynx after treatment (A, B) Follow-up laryngoscopic images taken after antifungal treatment, demonstrating marked improvement. The epiglottis and surrounding tissues exhibit reduced swelling, and the previously observed white patches have resolved.

Treatment was initiated with intravenous micafungin (MCFG) 100 mg daily and meropenem (MEPM) 1 g every eight hours, along with amphotericin B gargles (1 ml, three times daily). Inhaled medications were discontinued. The patient’s fever and sore throat improved by the following day. Neutrophil counts normalized by day 5, and oral intake improved. On the sixth day, upper gastrointestinal endoscopy showed near-complete resolution of white patches and reduced swelling (Figure 2A, 2B), with no signs of esophageal candidiasis. Candida albicans was identified from sputum cultures obtained at the initial visit, while blood cultures remained negative. MEPM was discontinued on day 8, MCFG on day 13, and amphotericin B gargles on day 18, following confirmed improvement via laryngeal fibroscopy. No relapses occurred after discharge.

## Discussion

Epiglottitis, a form of cellulitis affecting the epiglottis and surrounding tissues, can progress rapidly to life-threatening airway obstruction if not treated promptly. While fever and sore throat are primary symptoms, the presence of stridor, drooling, or a muffled voice should raise suspicion for this condition. Sore throat is a common complaint in outpatient settings, often resulting from viral upper respiratory infections. In this case, the patient presented with fever and sore throat during chemotherapy, and significant neutropenia detected in blood tests led to initial management as febrile neutropenia, potentially overlooking the severity of the sore throat. However, additional symptoms - such as an unusual muffled voice, difficulty swallowing due to pain, and stridor - indicated a more serious condition, necessitating urgent airway management and expedited diagnosis, suggesting possible epiglottitis rather than a simple viral infection.

A lateral neck X-ray in this case revealed a “thumb sign.” A retrospective study by Ng et al. involving 106 patients with Candida epiglottitis found that only 71 had undergone lateral neck X-rays, with 55 demonstrating the thumb sign [5]. While the thumb sign on a lateral neck X-ray can assist in diagnosis, its absence does not rule out epiglottitis [5]. The gold standard for diagnosing epiglottitis remains visual identification of epiglottic swelling through laryngoscopy.

Haemophilus influenzae is a well-known cause of epiglottitis, but it is rarely cultured from adult patients; many cases involve oral flora. Conversely, Candida epiglottitis, as seen in this case, is uncommon. Candida, an oral commensal fungus found in 20-50% of healthy individuals’ oral cavities, typically does not exhibit pathogenicity but can become pathogenic with systemic or local immune suppression [4]. Previous studies have confirmed the occurrence of Candida epiglottitis in immunocompromised children and adults. Risk factors include viral infections (especially cytomegalovirus), cytotoxic chemotherapy, neutropenia, acquired immunodeficiency syndrome, and extensive antibiotic and steroid use [2,6]. In a retrospective review of 93 patients from 1999 to 2016, Issa and Thomas found that inhaled steroids were closely linked to fungal infections in the larynx. Additional risk factors include oral steroids, nasal steroid sprays, broad-spectrum antibiotics, diabetes, smoking, exposure to radiation or chemotherapy, and gastroesophageal reflux disease [3]. Walsh and Gray reported three cases of epiglottitis in patients with neutropenia, indicating that Candida epiglottitis can signal a Candida bronchopulmonary infection or disseminated infection, with delayed treatment potentially being fatal [1]. Interestingly, Candida epiglottitis can also occur in healthy individuals without immunosuppression [6-8], particularly when local mucosal barriers are compromised by mechanical, chemical, or thermal factors.

Differential diagnoses for pharyngolaryngeal candidiasis include malignancies, leukoplakia, lichen planus, diphtheria, and tuberculosis [9]. Given the potential for malignancy, a biopsy of the affected area should not be delayed. However, Issa and Thomas argue that biopsy and scraping cultures are often unnecessary for diagnosing fungal laryngitis, as treatment frequently resolves the infection before biopsy results are available [3]. They advocate for high clinical suspicion and thorough laryngoscopy to eliminate the need for invasive diagnostic procedures, noting that Candida typically responds well to treatment. In this case, the diagnosis was based on clinical findings and endoscopic observation of white pseudomembrane formation and epiglottic swelling, alongside sputum culture, without biopsy. Although biopsy is not deemed necessary if treatment yields rapid improvement, early consideration of pathology and culture tests is advisable if there is no quick response to treatment. Screening tests for invasive Candida infections, such as β-D-glucan and Candida mannan antigen tests, were not elevated in this case, suggesting the absence of invasive Candida infection.

Treatment for oral pharyngeal candidiasis varies based on severity. For mild cases, clotrimazole troches or miconazole mucoadhesive buccal 50-mg tablets are recommended. For moderate to severe disease, oral fluconazole, 100-200 mg daily for seven to 14 days, is advised. In cases resistant to fluconazole, itraconazole, posaconazole, or amphotericin B deoxycholate oral suspension is recommended [10]. Despite negative blood cultures and β-D-glucan tests, the patient’s significant neutropenia, elevated CRP levels, and high fever raised concerns for possible Candida sepsis. An echinocandin (caspofungin: loading dose of 70 mg, then 50 mg daily; MCFG: 100 mg daily; anidulafungin: loading dose of 200 mg, then 100 mg daily) is recommended as initial therapy for candidemia in neutropenic patients [10]. In this case, the sore throat improved by the following day after gargling with amphotericin B syrup and administering intravenous MCFG 100 mg daily. This rapid response is consistent with other reported cases in the literature. Sharma et al. noted significant symptom resolution within two days in a young HIV-positive patient with Candida epiglottitis [11]. Similarly, Singh et al. reported symptom improvement within 12 hours of initiating oral antifungal treatment [6]. The swift response observed in our case and others suggests that with prompt and appropriate treatment, the prognosis for Candida epiglottitis is generally favorable. This underscores the importance of early recognition and targeted therapy in managing this condition.

## Conclusions

This report details a case of Candida epiglottitis in a patient receiving chemotherapy for small-cell lung cancer, coupled with inhaled steroid use, which led to neutropenia. While pharyngolaryngeal candidiasis is rare, it can become life-threatening if treatment is delayed. Thus, when immunocompromised patients or those with local mucosal damage present with a sore throat, this condition should be included in the differential diagnosis and managed appropriately. Clinicians must maintain a high index of suspicion for fungal etiologies in at-risk populations to facilitate timely intervention and improve patient outcomes.
